# Supplementary material for: Maternal Phylogenetic Relationships and Genetic Variation among Rare, Phenotypically Similar Donkey Breeds
Source: Genes (Basel). 2021 Jul 22;12(8):1109. doi: 10.3390/genes12081109 (PMC8392470; doi:10.3390/genes12081109)
Supplement: Supplementary file 1 [file genes-12-01109-s001.zip › genes-1291710-suppl-19_07_202.pdf]

Tabl S1. Microchip number of the samples successfully sequenced by breed and official breeding station.

#	Breed	Microchip	Haplotype	Official breeding station
1.	MF	13189021	1	Istituto Incremento Ippico - Regione Puglia
2.	MF	63415	3	Istituto Incremento Ippico - Regione Puglia
3.	MF	19184	5	Istituto Incremento Ippico - Regione Puglia
4.	MF	13189028	9	Istituto Incremento Ippico - Regione Puglia
5.	MF	4413183	10	Istituto Incremento Ippico - Regione Puglia
6.	MF	4414835	11	Istituto Incremento Ippico - Regione Puglia
7.	MF	63379	15	Istituto Incremento Ippico - Regione Puglia
8.	MF	63337	20	Istituto Incremento Ippico - Regione Puglia
9.	MF	91643	22	Istituto Incremento Ippico - Regione Puglia
10.	MF	91606	24	Istituto Incremento Ippico - Regione Puglia
11.	MF	63397	25	Istituto Incremento Ippico - Regione Puglia
12.	MF	94100003863333	30[s]	Istituto Incremento Ippico - Regione Puglia
13.	MF	94100001386334	30[s]	Istituto Incremento Ippico - Regione Puglia
14.	MF	941000014559394	34	Istituto Incremento Ippico - Regione Puglia
15.	MF	11573	36[s]	Istituto Incremento Ippico - Regione Puglia
16.	MF	36305	37[s]	Istituto Incremento Ippico - Regione Puglia
17.	MF	380271005004547	39	Istituto Incremento Ippico - Regione Puglia
18.	MF	13216391	41	Istituto Incremento Ippico - Regione Puglia
19.	MF	12040292	46	Istituto Incremento Ippico - Regione Puglia
20.	MF	100508589	47	Istituto Incremento Ippico - Regione Puglia
21.	MF	12040232	51[s]	Istituto Incremento Ippico - Regione Puglia
22.	MF	63369	51[s]	Istituto Incremento Ippico - Regione Puglia
23.	MF	12040558	51[s]	Istituto Incremento Ippico - Regione Puglia
24.	MF	12510	51[s]	Istituto Incremento Ippico - Regione Puglia
25.	MF	22936	51[s]	Istituto Incremento Ippico - Regione Puglia
26.	MF	393978	54	Istituto Incremento Ippico - Regione Puglia
27.	MF	91697	56	Istituto Incremento Ippico - Regione Puglia
28.	RG	941000013862001	4	Azienda agricola Chiara lo Cicero - Monti Nebrodi, Messina
29.	RG	12040354	6	Istituto Incremento Ippico - Regione Puglia
30.	RG	4590	8	Asinalat – Villafrati, Palermo
31.	RG	539497	12	Istituto Incremento Ippico - Regione Puglia
32.	RG	941000013861988	13	Azienda agricola Chiara lo Cicero - Monti Nebrodi, Messina
33.	RG	12040252	16	Istituto Incremento Ippico - Regione Puglia
34.	RG	985100007048356	17	Carabinieri distaccamento centro Forestale - Ragusa
35.	RG	7046	18	Asinalat – Villafrati, Palermo
36.	RG	1297789	19	Istituto Incremento Ippico - Regione Puglia
37.	RG	939000010009301	21	Asilat srl Asilandia – Milo, Catania
38.	RG	941000012764727	23	Carabinieri distaccamento centro Forestale - Ragusa
39.	RG	939000010006090	29	Asilat srl Asilandia – Milo, Catania
40.	RG	531688	36[s]	Istituto Incremento Ippico - Regione Puglia
41.	RG	380098100805226	37[s]	Carabinieri distaccamento centro Forestale - Ragusa
42.	RG	12040347	38	Istituto Incremento Ippico - Regione Puglia
43.	RG	941000013861952	42	Azienda agricola Chiara lo Cicero - Monti Nebrodi, Messina
44.	RG	380098100528952	43	Asilat srl Asilandia – Milo, Catania
45.	RG	981100000093854	44	Asilat srl Asilandia – Milo, Catania
46.	RG	941000013862011	48	Azienda agricola Chiara lo Cicero - Monti Nebrodi, Messina
47.	RG	4650	49	Asinalat – Villafrati, Palermo
48.	RG	12040252	51[s]	Istituto Incremento Ippico - Regione Puglia
49.	RG	1295563	55	Istituto Incremento Ippico - Regione Puglia
50.	CT	724090000013751	1	Fuives Centre Mundial de l'Ase Català Masia – Olvan, Barcelona
51.	CT	22380807	2	Istituto Incremento Ippico - Regione Puglia
52.	CT	724090000013754	14	Fuives Centre Mundial de l'Ase Català Masia – Olvan, Barcelona
53.	CT	72401114F000754	26	Fuives Centre Mundial de l'Ase Català Masia – Olvan, Barcelona
54.	CT	72401114F000760	27	Fuives Centre Mundial de l'Ase Català Masia – Olvan, Barcelona
55.	CT	724090000031426	28	Facultat de Medicina Veterinaria, U.A.B. - Barcelona
56.	CT	412D756A63	30[s]	Fuives Centre Mundial de l'Ase Català Masia – Olvan, Barcelona
57.	CT	724090000013759	31	Fuives Centre Mundial de l'Ase Català Masia – Olvan, Barcelona
58.	CT	72401114F000758	33	Fuives Centre Mundial de l'Ase Català Masia – Olvan, Barcelona

59.	CT	724090000013814	40[s]	Fuives Centre Mundial de l'Ase Català Masia – Olvan, Barcelona
60.	CT	412D56531F	40[s]	Fuives Centre Mundial de l'Ase Català Masia – Olvan, Barcelona
61.	CT	412D6D067E	40[s]	Fuives Centre Mundial de l'Ase Català Masia – Olvan, Barcelona
62.	CT	98510001033942	45	Fuives Centre Mundial de l'Ase Català Masia – Olvan, Barcelona
63.	CT	412B26457E	51[s]	Fuives Centre Mundial de l'Ase Català Masia – Olvan, Barcelona
64.	CT	724090000013813	52	Fuives Centre Mundial de l'Ase Català Masia – Olvan, Barcelona
65.	CT	985120022507249	53[s]	Fuives Centre Mundial de l'Ase Català Masia – Olvan, Barcelona
66.	CT	724090000013816	53[s]	Fuives Centre Mundial de l'Ase Català Masia – Olvan, Barcelona
67.	CT	72401114F000756	53[s]	Fuives Centre Mundial de l'Ase Català Masia – Olvan, Barcelona
68.	CT	724090000007329	53[s]	Fuives Centre Mundial de l'Ase Català Masia – Olvan, Barcelona
69.	PT	941000012767130	7	Azienda e Museo Agriforestale di San Matteo – Erice, Trapani
70.	PT	939000010007372	32	Azienda e Museo Agriforestale di San Matteo – Erice, Trapani
71.	PT	939000010006420	35	Azienda e Museo Agriforestale di San Matteo – Erice, Trapani
72.	PT	941000011408475	22	Azienda e Museo Agriforestale di San Matteo – Erice, Trapani
73.	PT	985100006936020	37[s]	Azienda e Museo Agriforestale di San Matteo – Erice, Trapani
74.	PT	941000012132951	51[s]	Azienda e Museo Agriforestale di San Matteo – Erice, Trapani
75.	PT	380098101357415	51[s]	Azienda e Museo Agriforestale di San Matteo – Erice, Trapani
76.	PT	985100006916533	51[s]	Azienda e Museo Agriforestale di San Matteo – Erice, Trapani
77.	CB		50	Azienda Agricola Orlando Prizzi/Palermo

[s] is for shared haplotypes. Istituto Incremento Ippico cooperate with Facoltà di Medicina Veterinaria Università degli Studi di Teramo (Fondo Rustico Chiareto) and Centro di Conservazione del Patrimonio Genetico dell'Asino della razza Martina Franca (Azienda agricola Russoli Crispiano). Istituto Incremento Ippico Regione Campania - Santa Maria Capua Vetere and Azienda Agricola Ciro Schirò Corleone – Monreale unfortunately have no successful sequenced samples. CB is for cross breed.

Table S5. Nucleotide frequencies per breed. A multivariate test of significance returned no differences in nucleotide composition among breeds (p = 0.98).

	**A**	**T(U)**	**C**	**G**
**MF**	38.42 (2.46)	31.51 (2.57)	21.12 (1.30)	8.95(2.72)
**RG**	38.28 (3.76)	31.58 (2.94)	21.12 (0.87)	9.02 (3.43)
**CT**	38.72 (1.13)	31.63 (0.95)	21.48 (0.71)	8.16 (0.65)
**PT**	39.1 (0.61)	31.58 (0.43)	21.43 (0.57)	7.89 (0.38)
**all**	38.37 (2.81)	31.62 (2.43)	21.18 (1.02)	8.83 (2.74)

In parenthesis standard deviation ±SD. The analysis was conducted in MEGA7.

Table S6. Maximum composite likelihood estimate of the pattern of nucleotide substitution. Each entry shows the probability of substitution (r) from one base (row) to another base (column). Rates of different transitional (A × G and C × T) substitutions are shown in bold and those of transversional (G × T and A × C) substitutions are shown in italics.

	**A**	**T**	**C**	**G**
MF transition/transversion rate ratios purines 4.617, pyrimidines 1.391; overall transition/transversion 1.008				
A	-	*6.41*	*4.29*	**8.4**
T	*7.81*	-	**5.97**	*1.82*
C	*7.81*	**8.91**	-	*1.82*
G	**36.06**	*6.41*	*4.29*	-
RG transition/transversion rate ratios purines 3.872, pyrimidines 0.95; overall transition/transversion 0.791				
A	-	*7.29*	*4.88*	**8.06**
T	*8.84*	-	**4.63**	*2.08*
C	*8.84*	**6.93**	-	*2.08*
G	**34.21**	*7.29*	*4.88*	-
CT transition/transversion rate ratios purines 0.985, pyrimidines 0; overall transition/transversion 0.125				
A	-	*12.85*	*8.73*	**3.27**
T	*15.73*	-	**0**	*3.32*
C	*15.73*	**0**	-	*3.32*
G	**15.49**	*12.85*	*8.73*	-
PT transition/transversion rate ratios purines, pyrimidines and overall transition/transversion is 0				

A	-	*15.79*	*10.71*	**0**
T	*19.55*	-	**0**	*3.95*
C	*19.55*	**0**	-	*3.95*
G	**0**	*15.79*	*10.71*	-

For simplicity, the sum of r values is made equal to 100. All positions containing gaps and missing data were eliminated. Evolutionary analyses were conducted in MEGA7.

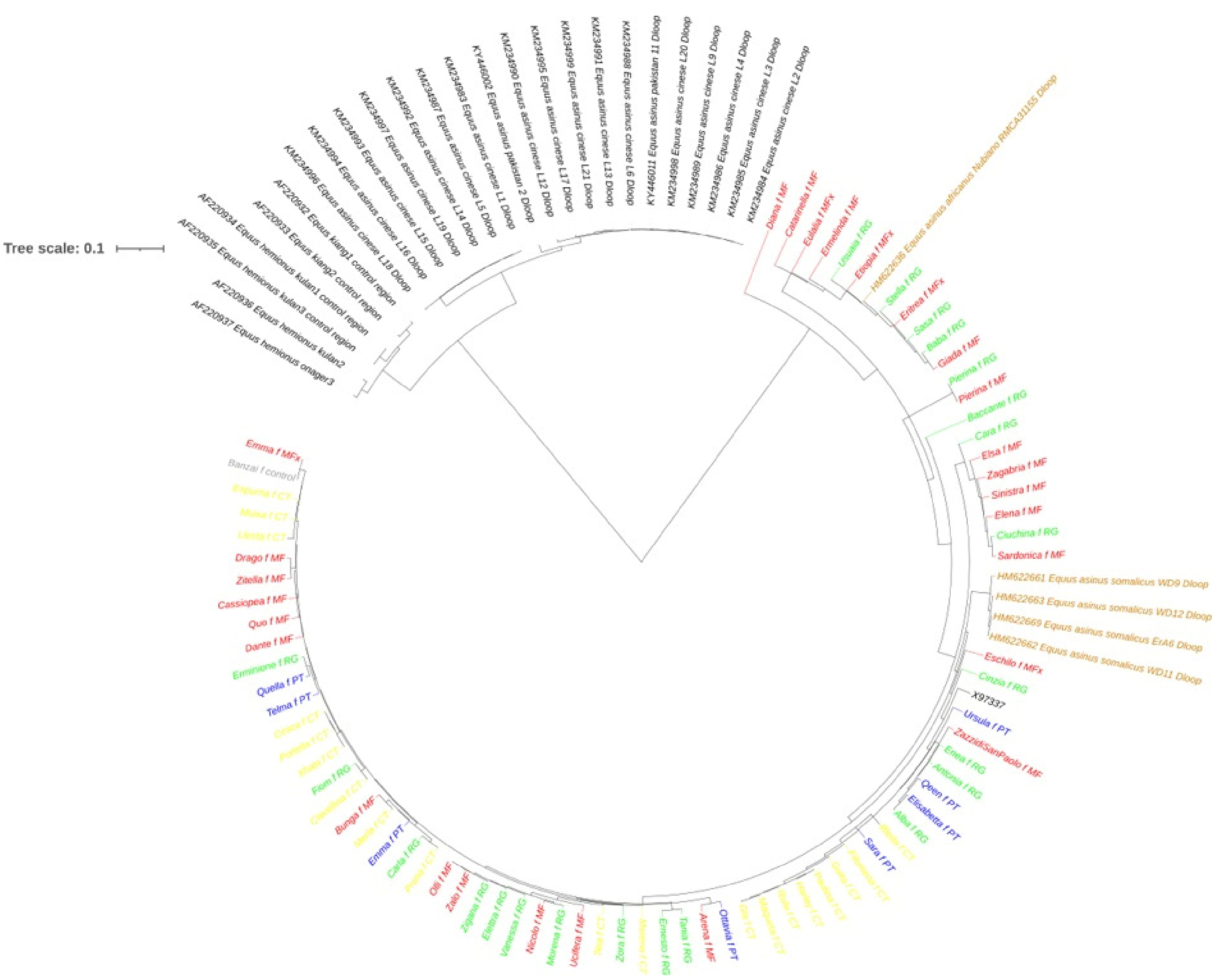

See 300dpi TIFF image also in a separate file

Supplementary Figure S1. All sequences were aligned with reference sequence GenBank X97337 and other similar sequences present in GenBank representative of African donkey lines Somalicus and Nubianus, Chinese ones and other Asiatic Equus kiang, E. hemionus and E. hemionus kulan.

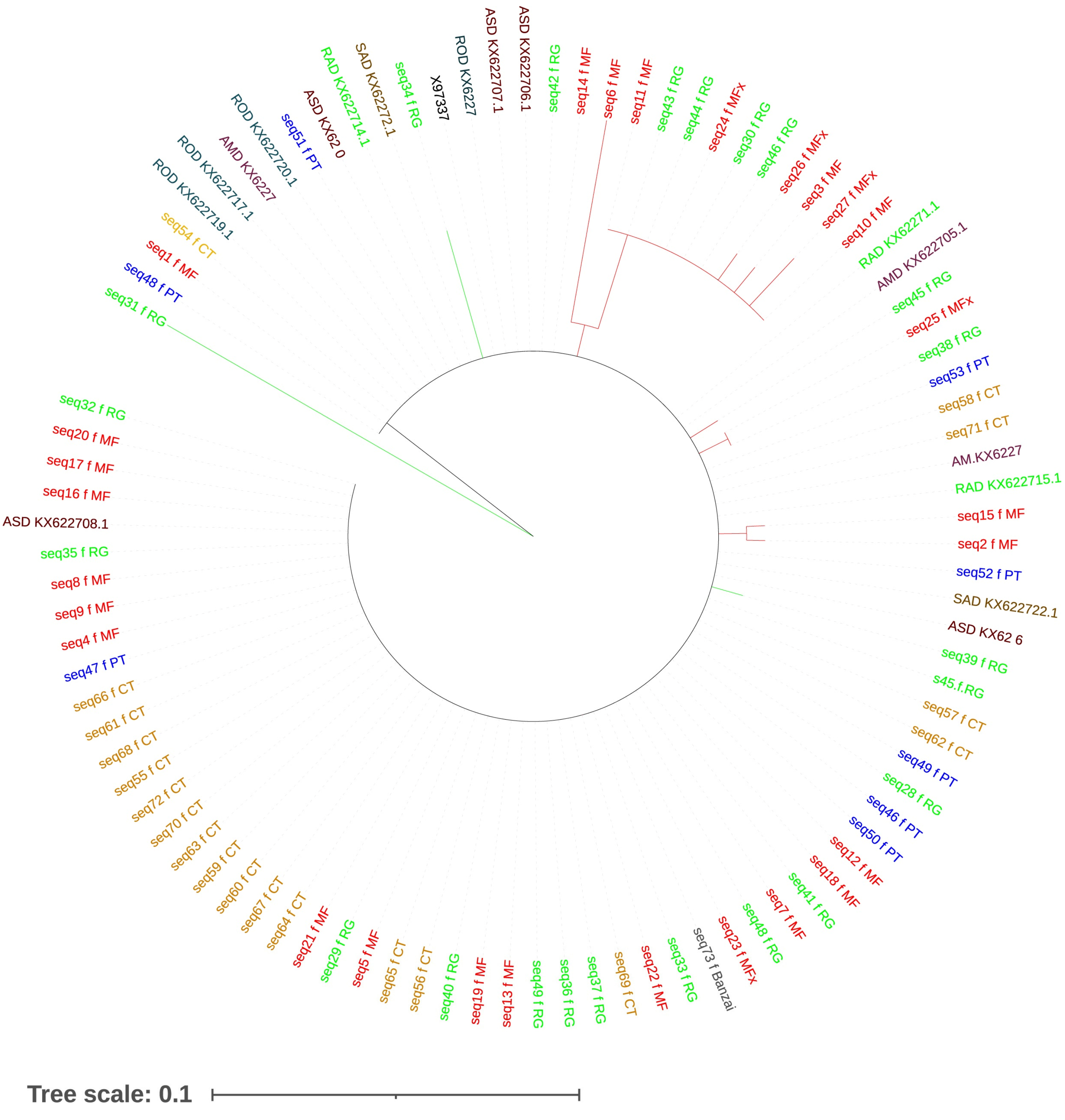

See 300dpi TIFF image also in a separate file

Supplementary Figure S2. The sequences aligned with the reference GenBank X97337 and other similar GenBank sequences representative Italian other breeds (as in ref. 42: Romagnolo donkey (ROD), Amiata donkey (AMD), Sar-dinian donkey (SAD), Asinara donkey (ASD), Ragusano donkey (RAD)).
